# Prognostication in Stargardt Disease Using Fundus Autofluorescence: Improving Patient Care

**DOI:** 10.1016/j.ophtha.2023.06.010

**Published:** 2023-11

**Authors:** Malena Daich Varela, Yannik Laich, Shaima Awadh Hashem, Omar A. Mahroo, Andrew R. Webster, Michel Michaelides

**Affiliations:** 1 Moorfields Eye Hospital, London, United Kingdom; 2 UCL Institute of Ophthalmology, University College London, London, United Kingdom; 3 Eye Center, Medical Center, Faculty of Medicine, University of Freiburg, Freiburg, Germany

**Keywords:** Autofluorescence, Electroretinogram, Genetics, Retina, Stargardt

## Abstract

**Purpose:**

To explore fundus autofluorescence (FAF) imaging as an alternative to electroretinography as a noninvasive, quick, and readily interpretable method to predict disease progression in Stargardt disease (STGD).

**Design:**

Retrospective case series of patients who attended Moorfields Eye Hospital (London, United Kingdom).

**Participants:**

Patients with STGD who met the following criteria were included: (1) biallelic disease-causing variants in *ABCA4*, (2) electroretinography testing performed in house with an unequivocal electroretinography group classification, and (3) ultrawidefield (UWF) FAF imaging performed up to 2 years before or after the electroretinography.

**Methods:**

Patients were divided into 3 electroretinography groups based on retinal function and 3 FAF groups according to the extent of hypoautofluorescence and retinal background appearance. Fundus autofluorescence images of 30° and 55° were reviewed subsequently.

**Main Outcome Measures:**

Electroretinography and FAF concordance and its association with baseline visual acuity (VA) and genetics.

**Results:**

Two hundred thirty-four patients were included in the cohort. One hundred seventy patients (73%) were in electroretinography and FAF groups of the same severity, 33 (14%) were in a milder FAF than electroretinography group, and 31 (13%) were in a more severe FAF than electroretinography group. Children < 10 years of age (n = 23) showed the lowest electroretinography and FAF concordance at 57% (9 of the 10 with discordant electroretinography and FAF showed milder FAF than electroretinography), and adults with adult onset showed the highest (80%). In 97% and 98% of patients, 30° and 55° FAF imaging, respectively, matched with the group defined by UWF FAF.

**Conclusions:**

We demonstrated that FAF imaging is an effective method to determine the extent of retinal involvement and thereby inform prognostication by comparing FAF with the current gold standard of electroretinography. In 80% of patients in our large molecularly proven cohort, we were able to predict if the disease was confined to the macula or also affected the peripheral retina. Children assessed at a young age, with at least 1 null variant, early disease onset, poor initial VA, or a combination thereof may have wider retinal involvement than predicted by FAF alone, may progress to a more severe FAF phenotype over time, or both.

**Financial Disclosure(s):**

Proprietary or commercial disclosure may be found in the Footnotes and Disclosures at the end of this article.

Stargardt disease (STGD) (Mendelian Inheritance in Man identifier, #248200) is the most common inherited retinal dystrophy worldwide, with an estimated prevalence of 1 in 6578 individuals.^1^, ^2^, ^3^ Stargardt disease was first described more than a century ago and occurs as a result of biallelic disease-causing variants in *ABCA4*, with > 1500 pathogenic variants reported to date.^4^^,^^5^ *ABCA4* encodes a transmembrane protein located in photoreceptor discs, responsible for translocating all-trans Retinal and its byproducts from inside the outer segment discs to the photoreceptor cytoplasm.^6^ It is inherited in an autosomal recessive pattern; however, because up to 10% of the population carries pathogenic variants in *ABCA4*, pseudodominance can also occur.^7^

Stargardt disease has a highly variable phenotype, with an age of onset ranging from < 10 years to the seventh decade, with incidence peaking in childhood, early adulthood, and, less frequently, late adulthood.^8^ The most common visual symptoms are central vision loss, delayed dark adaptation, and pericentral scotomas, and patients often become severely visually impaired 5 to 11 years after symptom onset.^9^, ^10^, ^11^

Retinal examination is typically characterized by macular atrophy and pisciform yellow deposits in the perimacula.^8^ Comprehensive investigations are important for early accurate clinical diagnosis and monitoring, including fundus autofluorescence (FAF) imaging, spectral-domain OCT, and electrophysiologic assessment.^12^ Several clinical classifications have been established to help assess disease severity and to correlate with genotype. Fundus autofluorescence-based categorization typically comprises 3 groups: the first with circumscribed decreased autofluorescence (DAF) at the fovea and a homogeneous background, the second with DAF at the macula and a heterogeneous background, and the third with multiple areas of DAF at or beyond the posterior pole.^13^, ^14^, ^15^, ^16^ This classification was previously used in a smaller cohort to correlate the different FAF groups with functional parameters such as best-correct visual acuity (BCVA), visual field results, and electroretinography findings.^17^

Electrophysiologic assessment is particularly helpful in providing better-informed advice on prognosis.^18^^,^^19^ A classification of 3 functional phenotypes based on electroretinography findings is well established: group 1 consists of severe pattern electroretinography abnormality (macular dysfunction) with normal full-field electroretinography (ffERG), group 2 consists of severe pattern electroretinography abnormality with additional generalized cone dysfunction on ffERG, and group 3 consists of severe pattern electroretinography abnormality with additional generalized cone and rod dysfunction on ffERG.^18^^,^^19^ A longitudinal electroretinography study confirmed the prognostic implications of the aforementioned electroretinography groups, with group 1 having the best prognosis, group 2 having an intermediate or variable prognosis, and group 3 having the worst prognosis.^18^^,^^19^ All patients with initial rod electroretinography involvement demonstrated clinically significant electrophysiologic deterioration, whereas only 20% of patients with normal ffERG findings at baseline showed clinically significant progression over time. These findings are supported by the association with genotype grouping (e.g., group 1 harboring milder variants, whereas group 3 is associated with a greater prevalence of null variants).^13^^,^^20^^,^^21^ Further analysis demonstrated that those with abnormal ffERG results also showed decreased BCVA and higher rate of scotoma and atrophy enlargement than those with normal ffERG results.^15^^,^^22^

Despite its usefulness in providing advice on prognosis in STGD, electroretinography testing is not (readily) available in many centers worldwide, it is challenging and time consuming to undertake testing reliably and interpret the results, it requires highly trained and dedicated personnel to perform testing and to provide reports, it has a high intersession variability, and it is often long and uncomfortable for patients. In direct contrast, FAF imaging, both widefield and posterior pole imaging, has none of these aforementioned limitations. In this study, FAF was explored as an alternative to electroretinography as a noninvasive, quick, cheap, and readily interpretable method available in most ophthalmology departments to predict disease progression.

## Methods

This study was a retrospective case series of patients who attended Moorfields Eye Hospital (London, United Kingdom) and received a diagnosis of STGD. Patients were identified through a clinical database search and had to meet the following criteria to be included in this study: (1) harboring biallelic disease-causing variants in *ABCA4*, (2) having undergone electroretinography testing at Moorfields Eye Hospital with an unequivocal report that allowed classification into an electroretinography group, and (3) undergoing ultrawidefield (UWF) FAF imaging up to 2 years before or after the electroretinography testing. Ultrawidefield FAF was chosen initially to be able to compare peripheral retinal imaging with peripheral retinal function (ffERG), given that the electroretinography prognostic groups are based on the extent of retinal involvement. Informed consent was obtained from all patients. Ethical approval was provided by the ethics committee of Moorfields Eye Hospital, and the study honored the tenets of the Declaration of Helsinki.

Patient electronic health care records were reviewed to retrieve relevant clinical information. Age at onset was defined as the age at which visual difficulties were first noted by the patient. Snellen visual acuities were recorded and converted to logarithm of the minimum angle of resolution (logMAR) values for the purpose of statistical analysis. Counting fingers vision was given a value of 1.98 logMAR, hand moments was given a value of 2.28 logMAR, light perception was given a value of 2.7 logMAR, and no light perception was given a value of 3.0 logMAR.^23^^,^^24^ When testing associations between groups and visual acuity (VA), only the right eye was considered to avoid a clustering effect. Patients were categorized using the World Health Organization visual impairment criteria that defines no or mild visual impairment as BCVA of ≤ 0.48 (Snellen 6/18 [meters], 20/60 [feet]), moderate impairment as BCVA of > 0.48 and ≤ 1.0 (6/60, 20/200), severe as BCVA of > 1.0 and ≤ 1.3 (3/60, 20/400), and blindness as BCVA of > 1.3.

**Figure 1:**
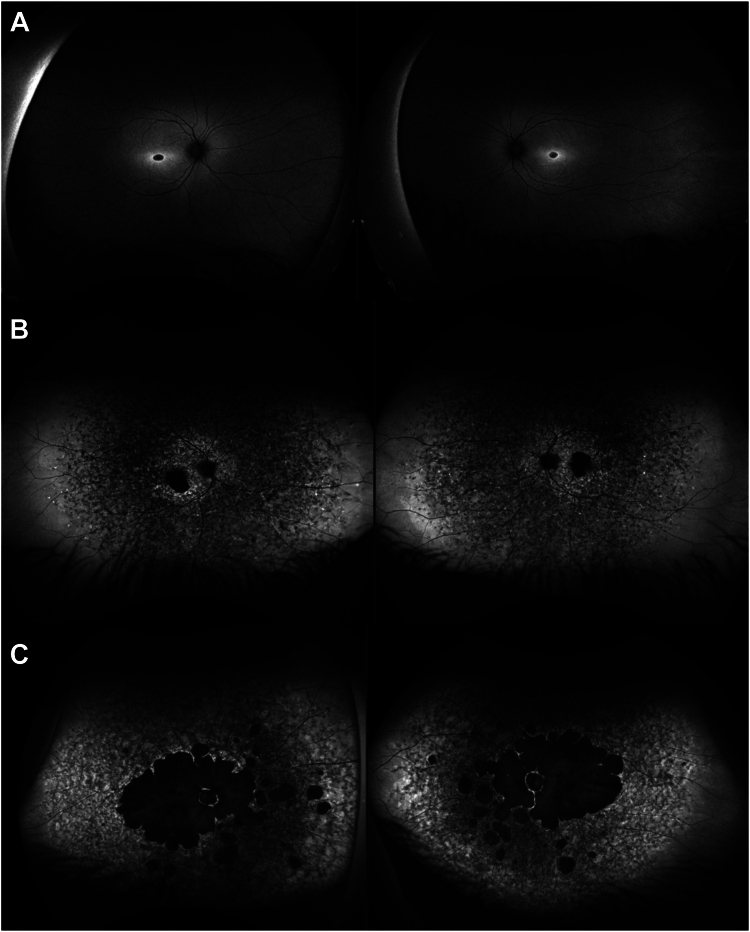
Ultrawidefield fundus autofluorescence images showing classification into 3 severity groups: (A) group 1 corresponds to an area of hypoautofluorescence at the fovea and a homogeneous background, (B) group 2 is characterized by ≥ 1 areas of hypoautofluorescence at the macula and a heterogeneous background, and (C) group 3 is represented by multiple areas of definitely decreased autofluorescence at the posterior pole extending beyond the vascular arcades and a heterogeneous background.

Ultrawidefield (green) FAF photography was carried out with Optos (Optos PLC). A subset of patients also underwent 30° and 55° (blue) FAF imaging (Heidelberg Spectralis, Heidelberg Engineering, Inc.). Based on previous work,^13^, ^14^, ^15^ individuals were classified into 3 FAF groups: group 1 included those with an area of hypoautofluorescence at the fovea and a homogeneous background, group 2 was characterized by an area(s) of hypoautofluorescence at the macula and a heterogeneous background, and group 3 was represented by ≥ 1 areas of definitely DAF at the posterior pole extending beyond the vascular arcades and a heterogeneous background (Fig 1).

Both pattern and ffERG testing were performed in all patients to determine the electroretinography group. Testing was carried out incorporating the International Society for Clinical Electrophysiology of Vision standards.^25^^,^^26^ Electroretinography groups corresponded to those described by Lois et al.^18^ Patients with electroretinography reports that were unclear or not definitive regarding electroretinography group were excluded (n = 14).

Genetic testing was performed using panel-based targeted next generation sequencing, entire exome sequencing, or entire genome sequencing. Where appropriate and when available, blood samples were obtained from parents or siblings to confirm segregation of proposed variants. Genotype grouping was performed according to the presence of ≥ 1 null variants that were assumed to result in a loss of function (nonsense, frameshift, splice site alteration, and exon deletion). Deep-intronic variants largely result in protein truncations; hence, they were also considered to be null.^27^

GraphPad Prism software version 8.0.2 (GraphPad Software) was used for statistical analysis. The threshold of significance was set at *P* < 0.05. *t* tests were used to assess parametric variables, chi-square tests assessed the relationship between categorical variables, and odds ratios were used to prove the association between 2 categories. Welch’s *t* test variation was used when the sample sizes were significantly different.

## Results

The final cohort that met all eligibility criteria comprised 234 patients who underwent electroretinography and FAF testing between 2012 and 2022 (median, 2018) at 33.7 ± 17.1 years of age (median, 32 years; range, 6–83 years; Table S1, available at www.aaojournal.org). Forty-three patients (18%) underwent assessments as children (< 17 years of age), and 191 patients (82%) underwent assessments as adults. One hundred forty-four patients (62%) underwent follow-up UWF FAF imaging, and 43 patients (18%) had undergone a previous electroretinography assessment.

**Figure 2:**
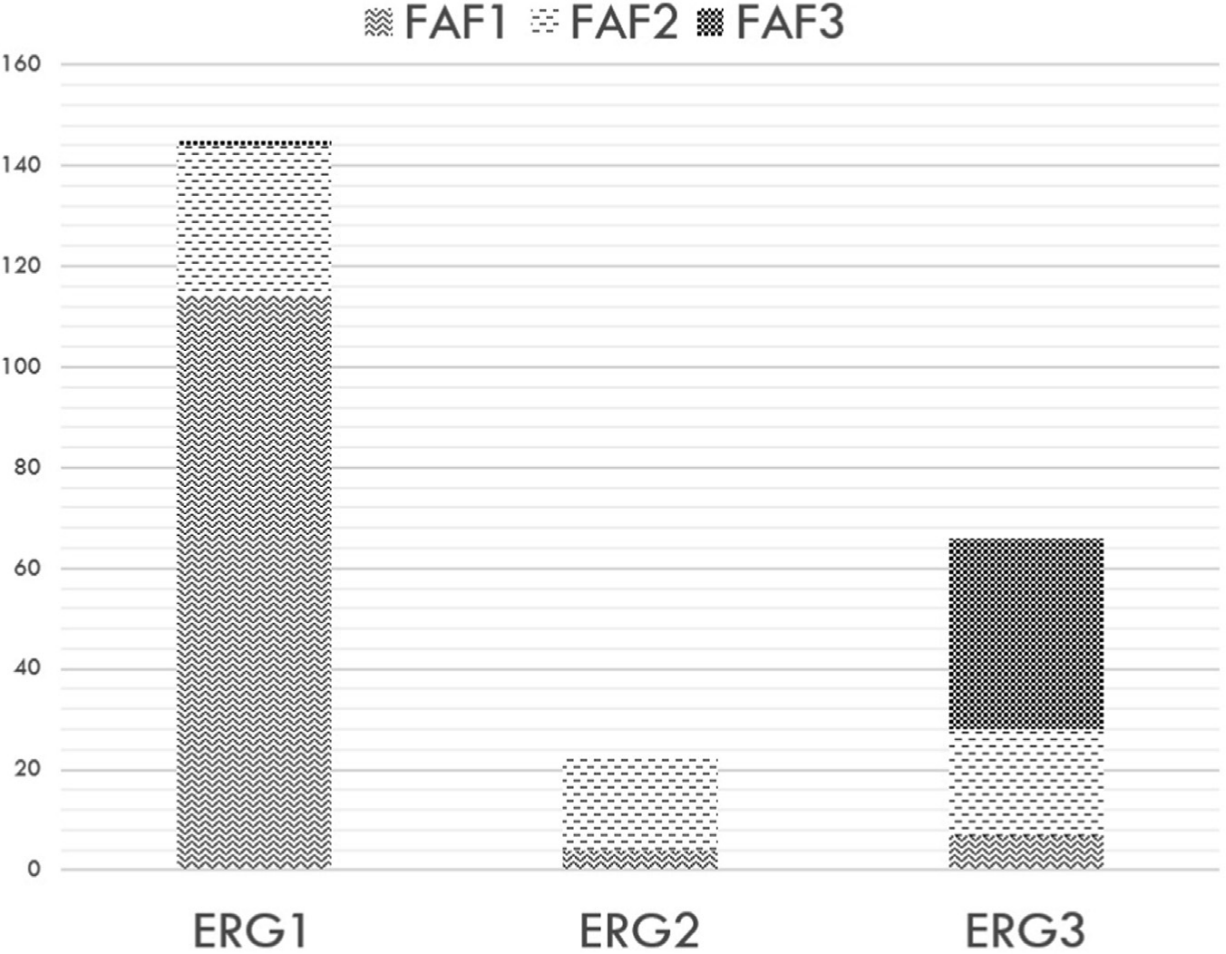
Bar graph showing electroretinography and fundus autofluorescence (FAF) groups in the cohort. Of the 234 patients included in total, 145 patients (62%) were in electroretinography group 1 (ERG1), 23 patients (10%) were in electroretinography group 2 (ERG2), and 66 patients (28%) were in electroretinography group 3 (ERG3). Of the 145 patients in ERG1, 114 patients (79%) were in FAF group 1 (FAF1), 30 patients (21%) were in FAF group 2 (FAF2), and 1 patient (1%) was in FAF group 3 (FAF3), with an overall ERG and FAF group match of 79%. Among the 23 patients in ERG2, 19 patients (83%) were in FAF2, and 4 patients (17%) were in FAF1, with an ERG and FAF group match of 83%. Of the 66 individuals in ERG3, 7 patients (11%) were in FAF1, 21 patients (32%) were in FAF2, and 38 patients (58%) were in FAF3, with an ERG and FAF match of 58%.

Considering electroretinography groups, 145 patients (62%) belonged to electroretinography group 1 (ERG1), 23 patients (10%) belonged to electroretinography group 2 (ERG2), and 66 patients (28%) belonged to electroretinography group 3 (ERG3; Table 2; Fig 2). Assessing UWF FAF, 125 patients (53%) belonged to FAF group 1 (FAF1), 70 patients (30%) belonged to FAF group 2 (FAF2), and 39 patients (17%) belonged to FAF group 3 (FAF3; Table 2; Fig 2). No significant differences were found in the age of the patients at the time of electroretinography and FAF between electroretinography groups (*P* = 0.49–0.96); however, patients in FAF3 were significantly older than those in FAF1 (*P* < 0.0001) and in FAF2 (*P* = 0.02). One hundred seventy patients (73%) were in the same electroretinography and FAF group, 33 patients (14%) were in a milder FAF than electroretinography group, and 31 patients (13%) were in a more severe FAF than electroretinography group. Those in a milder FAF than electroretinography group were significantly younger at the time of the assessment than those in a worse FAF than electroretinography group and those in the same FAF and electroretinography grouping (mean age, 19.9 years vs. 34.4 years and 31.9 years, respectively; *P* = 0.001).

**Table 2:** Cohort Characteristics

Group	Fundus Autofluorescence Group			Age (yrs)		Children	Adults		Missense Genotype	Null Genotype		Baseline Visual Acuity
	1	2	3	At Assessment	At Onset		Childhood Onset (Before 17 Years of Age)	Adult Onset (≥ 17 Years of Age)		1	≥ 2	
ERG1	114	30	1	33.7 ± 16.9	24.5 ± 14.5	24	24	85	72	64	9	0.6 ± 0.4
ERG2	4	19	0	34.8 ± 19.6	24.4 ± 19.1	5	6	8	8	9	6	0.7 ± 0.4
ERG3	7	21	38	33.5 ± 16.3	14.6 ± 11.7	14	28	12	23	30	13	1.1 ± 0.5
FAF1	∗	∗	∗	30.6 ± 16.4	22.5 ± 13.4	30	18	69	61	53	12	0.6 ± 0.4
FAF2	∗	∗	∗	34.7 ± 18.1	24.2 ± 18.3	10	21	29	33	28	8	0.7 ± 0.5
FAF3	∗	∗	∗	42 ± 13.9	14.8 ± 10.1	3	19	7	9	23	7	1.2 ± 0.6

ERG1 = electroretinography group 1; ERG2 = electroretinography group 2; ERG3 = electroretinography group 3; FAF1 = fundus autofluorescence group 1; FAF2 = fundus autofluorescence group 2; FAF3 = fundus autofluorescence group 3.

Data are presented as no. or mean ± standard deviation.

If ERG2 and ERG3 are combined to compare with ERG1, thereby to compare generalized retinal involvement versus isolated macular disease, respectively, 82% showed a matching electroretinography and FAF pattern: 78 of 89 patients (88%) in ERG2 and ERG3 and 114 of 145 patients (79%) in ERG1.

A significant association was found between the three electroretinography and FAF groups (*P* < 0.0001). Patients in ERG1 showed 51 times the odds of being in FAF1 compared with those in ERG3 and 18 times the odds compared with patients in ERG2. Patients in ERG2 showed 18 times the odds of being in FAF2 compared with being in ERG1 and 10 times the odds of someone in ERG3. Patients in ERG3 showed 195 times the odds of being in FAF3 compared with those in ERG1 and 31 times the odds compared with those in ERG2.

### Age and Disease Onset

Age at onset was available for 206 patients (88%) with a mean age of 21.9 ± 14.9 years (median, 18 years; range, 4–68 years). Forty-three patients were children (21%) with childhood onset, 58 patients were adults (28%) who were symptomatic before 17 years of age, and 105 patients were adults (51%) with symptoms onset at ≥ 17 years of age.

The most frequent groups in children (n = 43) were FAF1 (70%), ERG1 (56%), ERG3 (33%), and FAF2 (23%). In adults with childhood onset, the most common groups were ERG3 (48%), ERG1 (41%), FAF2 (36%), and FAF3 (33%). Finally, for adults with adulthood onset, the most common findings were ERG1 (81%), FAF1 (66%), FAF2 (28%), and ERG3 (11%).

Children in ERG3 were significantly younger than those in ERG1 (9 years of age vs. 11 years of age, respectively; *P* = 0.04). Children < 10 years of age (n = 23) showed the lowest electroretinography and FAF match, 57% (9 of the 10 with discordant electroretinography and FAF grouping showed milder FAF than electroretinography grouping), and adults with adult onset showed the highest with an 80% match. The highest mismatch was in ERG3 in children (4.6 times less FAF3 than expected), followed by ERG2 in adults (3.6 times more FAF2 than expected).

Patients in ERG3 showed a significantly earlier age at onset than those in ERG1 (14.6 years vs. 24.5 years; *P* < 0.0001), and patients in FAF3 also showed a significantly earlier age at onset when compared with those in FAF1 (14.8 years vs. 22.5 years; *P* = 0.001) and those in FAF2 (14.8 years vs. 24.2 years; *P* = 0.003). Those in a milder FAF than electroretinography group also showed significantly earlier age at onset compared with those with the same electroretinography and FAF grouping and those with a worse electroretinography than FAF grouping (13.9 years vs. 22.1 years and 28.9 years, respectively; *P* = 0.002 and *P* = 0.006, respectively). This pattern suggests that this discrepancy between FAF and electroretinography can be a potential feature of childhood-onset disease, in which functional impairment detectable by electroretinography precedes structural loss detectable by FAF.

### Electroretinography Group 1

Of the 145 patients in ERG1, 114 patients (79%) were in FAF1, 30 patients (21%) were in FAF2, and 1 patient (1%) was in FAF3, with an overall electroretinography and FAF group match of 79%. Twenty-four patients (17%) were children, 24 patients (17%) were adults with childhood onset, and 85 patients (59%) were adults with adult onset. No significant differences were found in age at onset (*P* = 0.18) or age at assessment (*P* = 0.07) between the matching (e.g., ERG1 and FAF1) and discordant groups. No differences were found regarding genotype, with 52% of the discordant group having at least 1 null variant versus 49% of the matching group and 48% of the discordant group having missense genotypes versus 50% of the matching group.

Twenty-one patients had undergone a previous electroretinography assessment 9 ± 4.6 years (range, 1–17 years) before the assessment included in the study; 20 patients were categorized in ERG1, and 1 patient was catergorized in ERG2. Ninety-two individuals underwent follow-up FAF after 3.6 ± 1.8 years (range, 1–10 years), and 7 patients (8%) progressed to a more severe FAF group over time, 4 of the latter being children < 10 years of age at the baseline visit for this study.

### Electroretinography Group 2

Among the 23 individuals in ERG2, 19 patients (83%) were in FAF2, and 4 patients (17%) were in FAF1, with an electroretinography and FAF group match of 83%. Three of the 4 discordant patients underwent assessments before 10 years of age. The remaining adult stayed in FAF group 1 until the latest follow-up 6 years after electroretinography. Five patients (22%) were children, 6 patients (26%) were adults with childhood onset, and 8 patients (35%) were adults with adult onset. Thirteen patients underwent follow up FAF after 4 ± 2 years (range, 1–7 years), and 2 adults progressed to a more severe FAF group. Five patients underwent a previous electroretinography assessment (5–16 years before), with no change between groups.

### Electroretinography Group 3

Of the 66 individuals in ERG3, 7 patients (11%) were in FAF1, 21 patients (32%) were in FAF2, and 38 patients (58%) were in FAF3, with an electroretinography and FAF group match of 58%. Six of the 7 patients in FAF1 underwent assessments before 10 years of age, and 2 of them underwent follow-up imaging at 12 and 14 years of age that showed progression to FAF2 and FAF3, respectively. Fourteen patients (21%) were children, 28 patients (42%) were adults with childhood onset, and 12 patients (18%) were adults with adult onset.

Thirty-nine patients underwent follow-up FAF after 3.5 ± 1.8 years (range, 1–7 years) and 5 patients progressed to a more severe FAF group. Fifteen patients underwent a previous electroretinography assessment (2–17 years before), 10 patients remained in the same group, 4 patients changed from ERG2 to ERG3 (1 child and 3 adults), and 1 adult changed from ERG1 to ERG3. One adult underwent a second electroretinography assessment 3 years after the electroretinography assessment used for this study and changed from ERG3 to ERG2 (and belonged in FAF2).

### Genetics

Dividing the cohort into FAF groups, a significantly higher proportion of missense genotypes versus at least 1 null was found in FAF1 and FAF2 compared with FAF3 (*P* = 0.009 and *P* = 0.005). Patients in FAF1 and FAF2 had 3 and 4 times the odds of having a missense genotype compared with those in FAF3, respectively. Considering electroretinography groups, significantly more missense genotypes versus 2 or more null were found in ERG1 compared with ERG2 (*P* = 0.02) and ERG3 (*P* = 0.003). Patients in ERG1 showed nearly twice (1.84) the odds of having a missense genotype compared with patients in ERG2 and ERG3.

Regarding genotypes, no significant differences were found in the percentage of missense and null variants among those with matching FAF and electroretinography groups, milder FAF than electroretinography groups, and worse FAF than electroretinography groups (*P* = 0.15).

The milder variant p.Gly1961Glu was seen primarily in patients with matching ERG1 and FAF1 groups (49 patients), being seen only once in ERG1 and FAF2, once in ERG2, and thrice in ERG3.^28^ The intronic variant c.5461–10T>C (previously associated with a more severe phenotype) was seen in 13 patients in ERG1, 11 patients in ERG3, and 2 patients in ERG2.^19^

### Baseline Visual Acuity

Patients in FAF3 showed significantly worse initial VA compared with those in FAF1 (*P* < 0.0001) and FAF2 (*P* < 0.0001). Similarly, those in ERG3 showed significantly worse initial VA compared with those in ERG1 (*P* < 0.0001) and those in ERG2 (*P* = 0.005). Children in ERG3 showed significantly worse VA compared with children in ERG1 (*P* = 0.005), despite being younger.

The group with milder FAF than electroretinography group showed significantly worse initial VA compared with those with worse FAF than electroretinography and those with the same electroretinography and FAF grouping (mean, 0.9 vs. 0.7 and 0.6; *P* = 0.002 and *P* = 0.03, respectively). Those with milder FAF than electroretinography group included the smallest proportion of patients with no or mild visual impairment (15% vs. 42% and 52% in those with matching electroretinography and FAF grouping and more severe FAF than electroretinography grouping, respectively), and consequently the largest proportion of patients with blindness (9% vs. 8% and 1%, respectively) and severe (12% vs. 6% and 0%, respectively) and moderate (64% vs. 44% and 45%, respectively) visual impairment.

### 30° and 55° Autofluorescence

One hundred forty-eight patients (63%) underwent both 30° and 55° FAF imaging concurrently with UWF FAF, 37 patients (16%) underwent only 55° and UWF FAF imaging, 41 patients (18%) underwent 30° and UWF FAF, and 8 patients (3%) underwent UWF imaging only. In 97% and 98% of patients, 30° and 55° FAF imaging, respectively, matched with the FAF group defined by UWF FAF. That is, when compared with UWF groups, FAF2 and FAF3 were not captured fully for 6 patients (3%) with 30° imaging and 3 patients (2%) with 55° imaging.

## Discussion

This study evaluated the largest cohort of patients from a single tertiary referral center with molecularly confirmed STGD and concurrent electrophysiologic assessment and FAF imaging (both UWF and 30° or 55°). The primary purpose was to assess if FAF imaging could be used to provide reliable information on disease extent and thereby inform prognostication by comparing it with the current gold standard of electroretinography testing and thereby inform patient management. We also explored any potential associations with various clinical and genetic parameters.

Electroretinography and FAF groups were associated significantly, with > 70% of patients having the same electroretinography and FAF group. If further simplified into isolated macular versus widespread retinal involvement, > 80% of patients showed matching electroretinography and FAF patterns. A similar likelihood was found of underestimating and overestimating severity of prognosis with FAF based on electroretinography data. A high correlation between electroretinography and FAF results also was described previously in a smaller cohort by Abalem et al.^17^ More than half of the cohort comprised adults with adult-onset STGD and belonged in ERG1 and FAF1, which was in keeping with previous reports.^18^^,^^29^

Only 10% of the cohort progressed to a more severe FAF phenotype during follow-up; this percentage is smaller than that of a previous study that analyzed fewer patients. Our study of a larger cohort may be more reflective of STGD behavior, but differences in cohort characteristics cannot be excluded.^14^ Previous reports also have described a progression in electroretinography groups over time, with 20% of patients in ERG1 and 40% in ERG2 progressing to more severe electroretinography groups.^19^ This was not captured in the cohort, but we found that 21% of the patients in ERG1 showed more severe FAF involvement. One possibility is that generalized electroretinography involvement (ERG2 and ERG3) may occur in these patients over time, and thereby FAF abnormalities have preceded functional changes in these patients; or this represents a true disconnect between these evaluations in a minority of patients. However, we also found that 17% of patients in ERG2 and 43% of ERG3 demonstrated a less severe FAF phenotype, and patients in FAF3 were significantly older than those in FAF1 and FAF2. This, in direct contrast, illustrates that functional changes may manifest before structural changes are visible, which would be the most common observation in inherited retinal disease.^30^

Children in ERG2 and ERG3 were younger than those in ERG1 and showed poorer FAF correlation. This may be the result of possible technical difficulties affecting this age group, as well as FAF changes indeed manifesting at an older age (4 of 7 children progressed to a more severe FAF group after turning 10 years of age). Childhood-onset STGD has been reported to be characterized by a greater rate of progression than adult-onset STDG.^31^, ^32^, ^33^, ^34^ Fundus autofluorescence “catching up” with electroretinography testing, with a high rate of atrophy development or enlargement, thereby would be in keeping.^14^

Patients with milder FAF severity than electroretinography were significantly younger at the time of assessment, demonstrated earlier age at onset, and were the largest baseline proportion of visually impaired patients when compared with those with the same and worse electroretinography and FAF groupings. Initial VA has been reported to have an impact on the rate of VA loss, with better baseline VA correlating with slowest change over time.^35^^,^^36^ Taken together, we observed that young patients in FAF1 with at least 1 null variant and with early disease onset and poor initial VA often demonstrate wider retinal involvement and progress to a more severe phenotype over time.

Missense genotypes were seen more often in milder phenotypes, as reported previously.^14^^,^^27^ The variant p.Gly1961Glu was the most common among patients with the least severe phenotype (ERG1 and FAF1), agreeing with previous reports that locate it at the milder end of the disease spectrum.^37^

Although peripheral retinal changes can occur in STGD and may change the FAF group in a minority of patients, we found that in 97% and 98% of patients, 30° and 55° FAF imaging matched with the FAF group defined by UWF FAF. This supports the potential use of Heidelberg FAF imaging not only for diagnosis and characterization of STGD but, moreover, for prognostication and counselling.

Several research efforts are ongoing currently, with multiple therapeutic approaches under development (e.g., drugs targeting lipofuscin formation, antisense oligonucleotides that rescue splice defects, gene supplementation, and stem cell-derived retinal pigment epithelium transplantation).^8^^,^^27^ Fundus autofluorescence represents a faster, cheaper, and widely available method of characterizing and stratifying patients that can be useful when assessing a patient’s suitability for a clinical trial and targeting patients most likely to respond.

Electrophysiologic testing is associated with notable intersession variability and low repeatability, which is why it is used rarely in clinical practice to monitor disease progression or in clinical trials to determine treatment response.^38^, ^39^, ^40^ In contrast, FAF imaging has proven to be a useful clinical monitoring tool, providing various quantitative parameters to assess longitudinally (including area of definitely DAF) and questionably DAF and their respective rates of change) and also functioning as an approved outcome measurement for interventional clinical trials.^41^^,^^42^ Ultrawidefield FAF imaging does not entail discomfort for the patients, not even needing dilating drops to acquire useful images. Heidelberg FAF ideally needs dilation, and testing can be uncomfortable. However, current techniques with reduced illuminance have shown good concordance with conventional FAF, thereby potentially avoiding patient discomfort.^43^ Current FAF limitations include the potential benefit of a standardized approach to quantify the spatial distribution of AF (i.e., quantitative FAF), not directly imaging retinal architecture (compared with OCT), and lack of availability of widefield FAF imaging devices.

This study’s limitations include its retrospective nature and data being acquired in a large-scale clinical context not suitable for autofluorescence quantification. These are offset for the most part by the large number of genetically confirmed individuals and the thorough multimodal evaluation.

In conclusion, UWF and 30° or 55° FAF imaging are excellent instruments from which we can infer to what extent the patient’s retina is affected. In most patients, particularly adults, this imaging will enable us to advise the patient regarding disease prognosis, primarily in terms of whether it will remain confined to the macula or affect the peripheral retina progressively. Patients assessed in early childhood (especially ≤ 10 years of age) who harbor at least 1 null variant, show poor initial VA, or both may have wider retinal involvement or progress to a more severe phenotype over time than that suggested by baseline FAF imaging. Therefore, careful counselling is required and, ideally where possible, International Society for Clinical Electrophysiology of Vision electroretinography if the most accurate advice on prognosis is desired at the earliest opportunity.
