# Supplementary material for: Prognostication in Stargardt Disease Using Fundus Autofluorescence: Improving Patient Care
Source: Ophthalmology. 2023 Nov;130(11):1182–90. doi: 10.1016/j.ophtha.2023.06.010 (PMC11108789; doi:10.1016/j.ophtha.2023.06.010)
Supplement: Table S1 [file mmc1.pdf]

Supplementary Table: Demographics, genetics, visual acuity, autofluorescence and electroretinogram characteristics of our the cohort.

ID	ERG Group	FAF Group	Baseline VA OD	Baseline VA OS	Baseline age	*ABCA4* allele 1 c.	*ABCA4* allele 1 p.	*ABCA4* allele 2 c.	*ABCA4* allele 2 p.	*ABCA4* allele 3 c.	*ABCA4* allele 3 p.	0 Null	1 Null	2 Null	Age of onset	Age of assessment	Change in ERG group	Change in FAF group
1	1	1	1.3	1.8	26	c.179C>T	p.Ala60Val	c.5882G>A	p.Gly1961Glu			1			15	26	NO	NO
2	1	1	0.76	0.72	9	c.1253T>C	p.(Phe418Ser)	c.4773+1G>T					1		8	9	NO	YES
3	1	1	0.2	0.2	59	c.5461-10T>C		c.466A>G	p.Ile156Val				1		50	69	NO	NO
4	1	2	0.5	0.2	59	c.4685T>C	p.Ile1562Thr	c.1222C>T	p.Arg408Ter			1			20	59	NO	NO
5	1	1	0.8	0.8	16	c.5222T>C	p.Leu1741Pro	c.5222T>C	p.Leu1741Pro			1			10	16	NO	NO
6	1	1	0.8	0.8	15	c.4889T>C	p.(Phe1630Ser)	c.5381C>A	p.(Ala1794Asp)			1			13	15	NO	NO
7	2	2	1	1	26	c.4253+4C>T		c.5908C>T	p.Leu1970Phe	c.5714+5G>A				1	15	29	NO	NO
8	1	1	0.6	0.6	24	c.5461-10T>C		c.5882G>A	p.Gly1961Glu				1		23	28	NO	NO
9	1	1	1	1	28	c.834delT		c.5882G>A	p.Gly1961Glu				1		25	30	NO	NO
10	1	1	0.8	0.7	11	c.5714+5G>A		c.5461-10T>C						1	10	11	NO	NO
11	1	2	0.8	1	26	c.2588G>C	p.Gly863Ala	c.5461-10T>C					1		NA	41	NO - FU after 6 years	NO
12	1	1	0.2	0.3	30	c.5281_5289del	p.Pro1761_Leu1763del	c.2588G>C	p.Gly863Ala			1			30	41	NO - FU after 6 years	NO
13	3	3	0.1	0.3	43	c.859-9T>C		c.6658C>T	p.(Gln2220*)					1	41	43	NO	NO
14	2	1	0.1	0.1	5	c.6729+5_6729+19del15		c.6729+5_6729+19del15						1	9	7	NO	NO
15	1	2	0.2	0.5	68	c.3289A>T	p.Arg2030Gln	c.6089G>A	p.Arg2030Gln			1			68	71	NO	YES
16	1	1	0.5	0.5	23	c.634C>T	p.(Arg212Cys)	c.5882G>A	p.(Gly1961Glu)			1			10	23	NO	NO
17	1	1	0	0	55	c.2588G>C	p.Gly863Ala	c.5461-10T>C					1		45	55	NO	NO
18	3	3	1.5	1.7	52	c.4469G>A	p.Cys1490Tyr	c.6118C>T	p.Arg2040Ter				1		5	52	NO	NO
19	1	1	1	1	36	c.4539+2028C>T		c.4069G>A	p.Ala1357Thr				1		NA	49	NO - FU after 16 years	NO
20	1	1	1	1	52	c.5882G>A	p.Gly1961Glu	c.4195G>A	p.Glu1399Lys			1			NA	66	NO	NO
21	1	1	0.8	0.8	37	c.4253+43G>A	c.6147+23C>T	c.4537delC	p.Gln1513ArgfsTer1					1	25	52	NO	NO
22	1	2	0	0	65	c.5461-1OT>C		c.6089G>A	p.(Arg203OGln)				1		66	71	NO	NO
23	2	1	1	1	39		p.Trp782*2		p.Ile478Thr		p.Asn247Thr		1		NA	51	NO - FU after 13 years	NO
24	3	3	CF	CF	23	c.4070C>T	p.(Ala1357Val)	c.3210_3211dup	p.(Ser1071Cysfs*14)				1		NA	35	NO - fu after 12 years	NO
25	3	3	1	1	47	c.5461-10T>C		c.2588G>C	p.(Gly863Ala)				1		NA	56	NO - FU after 16 years	NO
26	2	2	0.8	0.8	22	c.4469G>A	p.Cys1490Tyr	c.5196+1137G>A					1		NA	36	NO - FU after 16 years	NO
27	1	2	0.3	0.3	24	c.2588G>C	p.(Gly863AIa)	c.5461-1OT>C					1		NA	38	NO - FU after 16 years	NO
28	3	2	1.3	0.9	45	c.2588G>C	p.(Gly863Ala	c.6449G>A	p.(Cys2150Tyr)			1			34	57	YES - FU after 9 years - 2 to 3	NO
29	1	1	1	1	22	c.4793C>A	p.Ala1598Asp	c.4793C>A	p.Ala1598Asp			1			22	23	NO	NO
30	1	3	1.8	0.5	71	c.4685T>C	p.Ile1562Thr	c.5461-10T>C					1		NA	83	NO - FU after 12 years	NO
31	2	2	1	1	22	c.4601delT		c.1726G>C	p.Asp576His				1		20	36	NO - FU after 11 years	YES
32	3	3	1	1	31	c.4462T>C	p.Cys1488Arg	c.3638T>C	p.Leu1213Pro			1			NA	47	NO	NO
33	3	2	1	1	13	c.4139C>T	p.Pro1380Leu	c.5461-10T>C					1		12	25	NO	NO
34	2	2	1.3	1	37	c.634C>T	p.Arg212Cys	c.5019-13G>A		c.6730-46T>C				1	15	40	NO - FU after 9 years	NO
35	1	2	0.6	0.6	6	c.2966T>C	p.(Val989Ala)	c.5281_5289del	p.(Pro1761_Leu1763del)				1		NA	34	NO - FU after 17 years	NO
36	1	2	1	0.2	31	c.3322C>T	p.Arg1108Cys	c.5714+5G>A					1		NA	44	NO - FU after 14 years	NO
37	1	1	1	1	20	c.5882G>A	p.Gly1961Glu	c.161G>A	p.Gly54Asp			1			6	28	NO - FU after 17 years	NO
38	1	1	0.3	0.3	38	c.5461-10T>C		c.6320G>A	p.(Arg2107His)				1		38	42	NO	NO
39	3	3	0.4	0.6	34	c.4139C>T	p.Pro1380Leu	c.5196+1137G>A					1		27	43	NO - FU after 9 years	NO
40	3	3	1.8	1.5	43	c.6232A>C	p.Lys2078Gln	c.1292G>A	p.Trp431Ter				1		NA	43	NO	NO
41	3	3	0	0	46	c.1805G>A	p.R602Q	c.5113C>T	p.R1705W			1			NA	53	YES - FU after 7 years	NO
42	3	3	0	0	43	c.6079C>T	p.Leu2027Phe	c.5196+1G>A					1		30	50	NO - FU after 3 years	NO
43	1	1	0.6	0.6	14	c.2203delC	p.(Leu735CysfsTer52)	c.5882G>A	p.(Gly1961Glu)				1		14	14	NO	NO
44	2	2	0.8	0.8	40	c.4634+1delG		c.3369C>T	p.Ala1123Ala				1		11	47	NO - FU after 5 years	NO
45	1	1	0.7	1	34	c.2588G>C	p.Gly863Ala	c.1715G>A	p.Arg572Gln			1			26	33	NO	NO
46	2	2	0	0	63	c.656G>C	p.Arg219Thr	c.2588G>C	p.Gly863Ala			1			61	65	NO	NO
47	1	1	1	1	57	c.5882G>A	p.Gly1961Glu	c.93G>A	p.Trp31Ter				1		12	63	NO - FU after 6 years	NO
48	2	2	0.7	0.7	14	c.4577C>T	p.Thr1526Met	c.4577C>T	p.Thr1526Met			1			NA	19	NO	NO
49	2	2	0.9	0.8	14	c.1906C>T	p.Q636X	c.6817-2A>C					1		11	24	NO	NO
50	1	1	0.8	0.8	57	c.4253+43G>A		c.5603A>T	p. (Asn1868Ile)	c.1293G>T	p.Trp431Cys		1		40	56	NO	NO
51	1	1	0.2	0.2	28	c.6148G>C;	p.Val2050Leu	c.1819G>A	p.Gly607Arg			1			24	33	NO	NO
52	1	1	0.6	0.3	42	c.454C>T	p.Arg152Ter	c.2759G>A	p.Arg920His				1		36	42	NO	NO
53	2	2	0.6	0.8	19	c.4577C>T	p.Thr1526Met	c.5714+5G>A					1		18	22	NO	NO
54	3	2	0.3	0	56	c.5714+5G>A		c.3322C>T	p.Arg1108Cys				1		53	58	NO	NO
55	1	1	0.3	1	29	c.5882G>A	p.Gly1961Glu	c.160+5G>C					1		26	32	NO	NO
56	1	1	1	1	23	c.6229C>T	p.Arg2077Trp	c.5882G>A	p.Arg1961Glu			1			16	24	NO	NO
57	1	2	0.6	0.6	47	c.1804C>T	p.Arg602Trp	c.868C>T	p.Arg290Trp			1			19	30	NO	NO
58	1	1	1	0.2	34	c.1622T>C	p.Leu541Pro	c.3113C>T	p.Ala1038Val			1			33	35	NO	NO
59	1	1	0.4	0.3	49	c.5603A>T	p.Asn1868Ile	c.6118C>T	p.Arg2040Ter				1		46	52	NO	NO
60	1	2	0.8	0.8	18	c.3056C>T	p.Thr1019Met	c.2813T>C	p.Phe938Ser			1			16	21	NO	NO
61	1	2	0.6	0.6	16	c.4774-27T>C		c.5196+1137G>A						1	10	18	NO	NO
62	1	1	1	1	35	c.5882G>A	p.Gly1961Glu	c.2894A>G	p.Asn965Ser			1			NA	37	NO	NO
63	1	1	CF	CF	22	c.5882G>A	p.Gly1961Glu	c.1222C>T	p.Arg408Ter				1		21	23	NO	NO
64	1	1	0.4	0.4	19	c.6449G>A	p.Cys2150Tyr	c.5714+5G>A					1		16	21	NO	NO
65	3	2	0.9	1.1	10	c.3064G>A	p.Glu1022Lys	c.3064G>A	p.Glu1022Lys			1			9	10	NO	NO
66	1	1	1.1	1.3	51	c. 6729+5_6729+19del15		c.5882G>A	p.Gly1961Glu				1		25	51	NO	NO
67	1	1	0.9	0.9	6	c.6729+5_6729+19del15		c.6729+5_6729+19del15						1	6	6	NO	YES
68	1	1	0	1	31	c.6098T>G	p.Leu2033Arg	c.763C>T	p.Arg255Cys			1			31	31	NO	NO
69	1	1	HM	HM	34	c.6729+5_19del15		c.5882G>A	p.Gly1961Glu				1		20	35	NO	NO
70	1	1	0.2	0.2	25	c.3322C>T	p.Arg1108Cys	c.3482G>A	p.Arg1161His			1			25	26	NO	NO
71	1	1	0.2	0	26	c.5882G>A	p.G1961E	c.4793C>A	p.A1598D			1			NA	25	NO	NO
72	1	1	0.8	0.8	22	c.5461-10T>C		c.5882G>A	p.(Gly1961Glu)				1		15	22	NO	NO
73	1	2	0.2	0.3	19	c.3482G>A	p.(Arg116His)	c.4469G>A	p.(Cys1490Tyr)			1			16	20	NO	NO
74	1	1	0.2	0.4	44	c.3389T>C	p.Ile1130Thr	c.3389T>C	p.Ile1130Thr			1			NA	44	NO	NO
75	1	1	0.6	1	31	c.5882G>A	p.Gly1961Glu	c.5917delG	p.Val1973Ter				1		27	32	NO	NO
76	2	2	1	1	22	c.4539+2028C>T			p.Arg1640Trp		p.Trp1408Arg		1		13	22	NO	NO
77	1	2	0.8	0.8	27	c.5461-10T>C		c.2588G>C	p.Gly863Ala				1		27	28	NO	NO
78	1	2	0.2	0.2	9	c.3056C>T	p.Thr1019Met	c.4326C>A	p.Asn1442Lys			1			10	9	NO	NO
79	1	1	1	1	34	c.5882G>A	p.Gly1961Glu	c.5917delG	p.Val1973Ter				1		18	33	NO	NO
80	1	1	0.8	0.8	46	c.2588G>C	p.(Gly863Ala)	c.3814-3C>A					1		38	46	NO	NO
81	1	1	1.1	0.4	39	c.634C>T	p.Arg212Cys	c.5882G>A	p.Gly1961Glu			1			38	39	NO	NO
82	3	1	0.8	0.8	10	c.5917delG	p.Val1973Ter	c.5917delG	p.Val1973Ter					1	NA	10	NO	NO
83	1	1	0.4	1	31	c.3113C>T	p.Ala1038Val	c.1622T>C	p.Leu541Pro			1			22	31	NO	NO
84	1	1	0.4	0.4	18	c.2588G>C	p.Gly863Ala	c.5603A>T	p.Asn1868Ile			1			15	21	NO	NO

85	1	1	1	1	66	c.3260A>G	p.Glu1087Gly	c.5693G>A	p.Arg1898His			1			31	66	NO	NO
86	1	1	1.2	0.2	36	c.161G>C	p.Cys54Ser	c.1592A>G	p.Glu531Gly			1			32	35	NO	NO
87	3	3	1.5	1.5	40	c.4139C>T	p.Pro1380Leu	c.3259G>A	p.Glu1087Lys			1			11	40	NO	NO
88	1	1	1	1	34	c.5882G>A	p.Gly1961Glu	c.3064G>A	p.Glu1022Lys			1			19	34	NO	NO
89	1	1	0	0	53	c.5603A>T	p.Asn1868Ile	c.5461-10T>C		c.2588G>C	p.Gly863Ala		1		53	54	NO	NO
90	1	2	1	1.1	24	c.2690C>T	p.Thr897Ile	c.5413A>G	p.Asn1805Asp			1			13	24	NO	NO
91	1	1	0	0	29	c.6079C>T	p.Leu2027Phe	c.1519G>T	p.Asp507Tyr			1			29	29	NO	NO
92	1	1	0.2	0.4	59	c.3259G>A	p.Glu1087Lys	c.5882G>A	p.Gly1961Glu			1			58	59	NO	NO
93	3	1	1	1	7	c.6729+5_6729+19delGTTGGCCCTGGGGCA		c.6729+5_6729+19delGTTGGCCCTGGGGCA						1	7	7	NO	NO
94	1	1	0.8	0.8	19	c.4139C>T	p.Pro1380Leu	c.4594G>A	p.Asp1532Asn			1			18	19	NO	NO
95	1	1	0.7	0.7	9	c.4537dupC	p.(Gln1513ProfsTer42)	c.3113C>T	p.(Ala1038Val)				1		9	8	NO	NO
96	3	3	1.1	0.4	12	c.4537dupC	p.(Gln1513ProfsTer42)	c.3113C>T	p.(Ala1038Val)	c.1622T>C	p.(Leu541Pro)		1		12	12	NO	NO
97	1	1	0.8	0.8	42	c.5882G>A	p.Gly1961Glu	c.3322C>T	p.Arg1108Cys			1			40	43	NO	NO
98	1	1	0.4	0.4	63	c.3210_3211dup	p.Ser1071Cysfs*14	c.5603A>T	p.Asn1868Ile				1		58	62	NO	NO
99	1	1	0.3	0.8	51	c.6729+5_6729+19del		c.5882G>A	p.(Gly1961Glu)	c.448G>A	p.(Glu150Lys)		1		51	51	NO	NO
100	3	3	1.8	1.8	47	c.666_678delAAAGACGGTGCGC		c.634C>T	p.R212C				1		9	50	NO	NO
101	3	3	1.8	1.8	54	c.5461-10T>C		c.1715G>A	p.(Arg572Gln)	c.2588G>C	p.(Gly863Ala)		1		14	53	NO	NO
102	3	3	0.8	1	45	c.5714+5G>A		c.5461-10T>C						1	15	49	NO	NO
103	3	3	1	1	45	c.4981delC	p.Leu1661Terfs	c.3322C>T	p.Arg1108Cys				1		9	45	NO	NO
104	1	2	0	0	65	c.6079C>T	p.Leu2027Phe	c.5714+5G>T					1		65	64	NO	NO
105	3	3	1	1.3	27	c.5196+1137G>A		c.293A>G	p.(Asn98Ser)				1		NA	41	YES - FU after 18 years, 1 to 3	NO
106	3	3	1.3	1	20	c.4773G>C	p.(Gly1591=)	c.4139C>T;	p. (Pro1380Leu)			1			10	36	YES - FU after 17 years, 1 to 3	NO
107	3	3	CF	CF	34	c.2894A>G	p.Asn965Ser	c.6158G>A	p.Trp2053Ter				1		7	51	NO - FU after 10 years	NO
108	3	3	CF	1	22	IVS5461-10T>C		c.6079C>T	p.L2027F				1		NA	32	NO	NO
109	3	3	0.7	0.7	43	c.5461-10T>C		c.5714+5G>A						1	16	43	NO	NO
110	1	1	0.2	0.2	50	c.5882G>A	p.G1961E	c.3392_3393delinsG	p.Ala1131GlyfsTer17				1		50	58	NO - FU after 5 years	NO
111	1	2	1	1	36	c.6320G>A	p.Arg2107His	c.4919G>A	p.Arg1640Gln			1			NA	49	NO	NO
112	3	3	0.4	0.4	8	c.3081T>G	p.Y1027X	c.3081T>G	p.Y1027X					1	8	2	NO - FU after 13 years	NO
113	3	2	1	1	26	c.4469G>A	p.Cys1490Tyr	c.3197T>G	p.Met1066Arg			1			NA	28	NO	NO
114	3	2	CF	CF	20	c.2092T>C	p.Cys698Arg	c.2092T>C	p.Cys698Arg			1			7	20	NO - FU after 4 years	YES
115	1	2	1	0.8	13	c.5882G>A	p.Gly1961Glu	c.634C>T	p.Arg212Cys			1			12	24	NO	no
116	1	2	0.4	0.4	36	c.5714+5G>A		c.5761G>A	p.Val1921Met				1		32	45	NO - FU after 7 years	NO
117	1	1	0.8	0.3	23	c.5461-10T>C		c.4253+43G>A						1	16	34	YES - FU after 10 years, 2 to 1	NO
118	3	3	1.3	1.3	12	c.2588G>C	p.Gly863Ala	c.3364G>A	p.Glu1122Lys	c.6088C>T	p.R2030X		1		NA	26	NO	NO
119	3	3	CF	0.6	51	c.4577C>T	p.(Thr1526Met)	c.5714+5G>A					1		18	61	NO	NO
120	3	3	0.3	0.9	14	c.6119G>A	p.Arg2040Gln	c.618C>G	p.Ser206Arg			1			NA	26	YES - FU after 12 years, 2 to 3	NO
121	2	2	0.8	0.6	35	c.93G>A	p.Trp31X	c.71G>A	p.Arg24His				1		25	38	NO	NO
122	1	2	0.3	0.4	17	c.4918C>T	p.Arg1640Trp	c.2588G>C	p.Gly863Ala	c.5603A>T	p.Asn1868Ile	1			17	30	NO	no
123	3	2	1	1	11	c.2861A>C	p.Y954S	IVS5461-10T>C					1		NA	19	NO	YES
124	1	1	0.8	1	43	c.5882G>A	p.(Gly1961Glu)	c.5917delG	p.(Val1973Ter)				1		25	46	NO	no
125	1	1	0.9	0.9	38	c.2588G>C	p.Gly863Ala	c.4139C>T	p.Pro1380Leu			1			22	44	NO - FU after 7 years	NO
126	3	3	1.8	1.3	24	c.4234C>T	p.Gln1412Ter	c.4919G>A	p.Arg1640Gln				1		4	26	NO	no
127	1	2	1	1	35	c.2827C>T	p.Arg943Trp	c.4577C>T	p.Thr1526Met			1			22	41	NO - FU after 9 years	no
128	1	1	0	0	49	c.443-453T>C		c.768G>T	p.Val256=				1		52	52	NO	no
129	1	2	1.5	CF	33	c.4050G>C	p.Leu1350Leu	c.571-1G>T		c.5087G>A	p.Ser1696Asn		1		20	43	NO - FU after 10 years	NO
130	3	3	1.3	1.8	40	c.4225A>G	p.Ile1409Val	c.5461-10T>C					1		9	40	NO	NO
131	1	1	0.4	0.4	18	c.2588G>C	p.Gly863Ala	c.2041C>T	p.Arg681Ter				1		16	25	NO	NO
132	1	1	0.8	0.4	36	c.5603A>T	p.(Asn1868Ile)	c.6079C>T	p.(Leu2027Phe)	c.4253+43G>A			1		9	39	NO	no
133	1	2	0.5	0.5	12	c.1622T>C	p.Leu541Pro	c.3113C>T	p.Ala1038Val	c.5714+5G>A			1		11	14	NO	NO
134	1	1	0.4	0.4	42	c.5603A>T	p.Asn1868Ile	c.4326C>A	p.Asn1442Lys			1			40	52	NO - FU after 8 years	NO
135	1	1	0.4	1	33	c.1648G>T	p.Gly550Ter	c.5882G>A	p.Gly1961Glu				1		25	36	NO	NO
136	3	3	1.8	1.8	48	c.3259G>A	p.Glu1087Lys	c.3835_3840delTCTGAT		c.519G>T	p.Asp507Tyr		1		10	51	NO	NO
137	1	1	1	1	31	c.5882G>A	p.Gly1961Glu	c. 5917delG	p.Val1973Ter				1		15	35	NO	NO
138	3	3	1.5	1.5	27	c.3064G>A	p.Glu1022Lys	c.3064G>A	p.Glu1022Lys			1			7	32	NO	NO
139	1	1	0.3	0.3	8	c.2588G>C	p.Gly863Ala	c.5161_5162delAC					1		8	16	NO - FU after 7 years	NO
140	1	1	1.5	0.5	57	c.3098del	p.(Lys1033Serfs*51)	c.5603A>T	p.(Asn1868Ile)				1		43	59	NO	NO
141	3	2	0.8	0.9	11	c.4326C>A	p.Asn1442Lys	c.5461-10T>C					1		7	13	NO	NO
142	1	1	0.3	0.2	35	c.5882G>A	p.Gly1961Glu	c.3197T>G	p.Met1066Arg			1			33	37	NO	NO
143	3	2	1.8	1.3	39	c.5714+5G>A	c.3292C>T p.Arg1098Cys	c.488_491delTGAC	p.Leu163HisfsTer18					1	11	41	NO	NO
144	1	1	0.3	0.4	26	c.6319C>T	p.Arg2107Cys	c.3322C>T	p.Arg1108Cys			1			24	28	NO	no
145	1	1	0.8	0.2	46	c.5882G>A	p.Gly1961Glu	c.4256T>C	p.Met1419Thr	c.6445C>T	p.Arg2149Ter		1		41	48	NO	NO
146	1	1	1	1	8	c.4577C>T	p.Thr1526Met	c.4139C>T	p.Pro1380Leu			1			5	9	NO - FU after 2 years	yes
147	3	1	0.9	0.9	7	c.214G>A	p.gly72Arg	c.214G>A	p.gly72Arg			1			7	7	NO	YES
148	1	2	0.4	0.4	35	c.583C>T	p.Arg195Trp	c.6119G>A	p.Arg2040Gln	c.1938-637A>G			1		32	36	NO	no
149	1	1	0.5	0.5	9	c.3322C>T	p.Arg1108Cys	c.1253T>C	p.Phe418Ser			1			7	9	NO	no
150	3	2	1.5	1.5	29	c.3064G>A	p.Glu1022Lys	c.3064G>A	p.Glu1022Lys			1			9	29	NO	no
151	1	1	0.3	0.2	14	c.161G>A	p.Cys54Tyr	c.5882G>A	p.Gly1961Glu			1			13	14	NO	no
152	3	2	0.3	1	51	c.4537dupC	p.Gln1513ProfsTer42	c.455G>A	p.Arg152Gln				1		50	53	NO	no
153	3	2	0	0	50	c.2560G>T	p.Ala854Ser	c.1928T>G	p.Val643Gly	c.4918C>T p.Arg1640Trp	c.4222T>C p.Trp1408Arg	1			33	49	NO	NO
154	3	2	1.2	1.2	22	c.1648G>A	p.Gly550Arg	c.6416G>C	p.Arg2139Pro			1			10	21	NO	YES
155	1	2	0.3	1	33	c.3113C>T	p.(Ala1038Val)	c.4978C>T	p.(Pro1660Ser)			1			31	24	NO	no
156	1	1	0.8	0.8	21	c.4328G>A	p.Arg1443His	c.1906C>T	p.Gln636*				1		21	21	NO - FU after 6 years	no
157	1	1	1	1	25	c.3210_3211dup	p.(Ser1071Cys*14)	c.6320G>A	p.(Arg2107His)				1		23	26	NO	no
158	1	1	0.2	CF	16	c.6148-698G>A		c.5196+323A>C						1	16	15	NO	no
159	1	1	0.8	0.8	18	c.5051T>A	p.Ile1684Asn	c.4469G>A	p.Cys1490Try			1			13	21	NO	NO
160	3	1	0.8	0.8	6	c.5846delG	p.(Gly1949AlafsTer25)	c.5846delG	p.(Gly1949AlafsTer25)					1	6	7	NO	NO
161	2	1	0.8	0.9	8	c.1957C>T	p.Arg653Cys	c.1957C>T	p.Arg653Cys			1			8	8	NO	YES
162	3	3	1.3	1	42	c.3322C>Tp.(Arg1108Cys)		c.1906C>T	p.(Gln636*)				1		38	42	NO	no
163	1	1	1	0.8	6	c.1804C>T	p.Arg602Trp	c.5196+1G>A					1		5	6	NO - FU after 1 year	NO
164	3	3	1.8	1.7	18	c.6729+5_6729+19del		c.6729+5_6729+19del						1	5	24	NO	NO
165	1	1	0.3	0.4	19	c.4139C>T	p.Pro1380Leu	c.1715G>A	p.Arg572Gln	c.2588G>C	p.Gly863Ala	1			18	20	NO	NO
166	1	1	0.2	0.2	19	c.1715G>A	p.Arg572Gln	c.2588G>C	p.Gly863Ala	c.4139C>T	p.Pro1380Leu	1			18	20	NO	NO
167	3	3	1.3	1.3	36	c.6089G>A	p.Arg2030Gln	c.2609C>T	p.Pro870Leu	c.1A>G	p.Met1Val	1			6	35	NO	NO
168	2	2	0.4	0.3	38	c.655A>T	p.(Arg219*)	c.3113C>T	p.(Ala1038Val)				1		38	38	NO	NO
169	3	2	1.3	1.3	30	c.1622T>C	p.Leu541Pro	c.2588G>C	p.Gly863Ala	c.3113C>T	p.Ala1038Val	1			10	33	NO	NO
170	3	3	1.3	1.3	55	c.1343T>A	p.Met4484Lys	c.6305A>G	p.Asp2102Gly			1			37	55	NO	NO
171	1	1	1	1	39	c.4577C>T	p.Thr1526Met	c.5882G>A	p.Gly1961Glu			1			9	38	NO	NO

172	3	3	1.8	1.8	61		p.Arg1097Ter	c.4793C>A	p.Ala1598Asp				1		15	60	NO	NO
173	1	1	1.5	0.4	23	c.2588G>C	p.Gly863Ala	c.1381A>T	p.Lys461*				1		9	21	NO	NO
174	3	2	1	1.1	9	c.1622T>C	p.Leu541Pro	c.3113C>T	p.Ala1038Val	c.4469G>A	p.Cys1490Tyr	1			9	9	NO	NO
175	1	1	0.3	0.3	14	c.4873C>A	p.His1625Asn	c.5882G>A	p.Gly1961Glu			1			9	13	NO	NO
176	1	2	0.8	0.4	37	c.2588G>C	p.Gly863Ala	c.331_332del	p.Glu111Thrfs*49				1		28	36	NO	no
177	1	1	0.6	0.6	27	c.4793C>A	p.Ala1598Asp	c.4793C>A	p.Ala1598Asp			1			18	27	NO	no
178	3	1	0.8	1	30	c.5882G>A	p.Gly1961Glu	c.6088C>T	p.Arg2030Ter				1		NA	30	NO	no
179	1	1	0.2	0	25	c.4139C>T	p.Pro1380Leu	c.1715G>A	p.Arg572Gln	c.2588G>C	p.Gly863Ala	1			25	25	NO	no
180	3	2	1	1	24	c.1917C>A	p.Tyr639Ter	c.6079C>T	p.Leu2027Phe				1		9	24	YES - FU after 3 years, 3 to 2	no
181	1	1	0.8	0.8	24	c.5603A>T	p.Asn1868Ile	c.5461-10T>C	c.2588G>C		p.Gly863Ala		1		8	25	NO	no
182	1	1	0.4	0.2	20	c.3814-2A>T		c.5882G>A	p.Gly1961Glu				1		11	20	NO	no
183	1	1	1	1	66	c.4539+2028C>T		c.5882G>A	p.Gly1961Glu						35	66	NO	no
184	1	1	0.5	0.4	26	c.859-9T>C		c.6658C>T	p.Gln2220Ter					1	24	26	NO	yes
185	1	1	0.4	0.2	33	c.6721C>G	p.(Leu2241Val)	c.4469G>A	p.(Cys1490Tyr)			1			32	34	NO	yes
186	1	1	0.8	0.8	24	c.1622T>C	p.Leu541Pro	c.5882G>A	p.Gly1961Glu	c.3113C>T	p.Ala1038Val	1			24	24	NO	no
187	1	1	0.2	0.2	48	c.5882G>A	p.Gly1961Glu	c.6449G>A	p.Cys2150Tyr			1			45	47	NO	no
188	3	1	0.9	0.8	9	c.2963T>C	p.(Leu988Pro)	c.93G>A	p.(Trp31Ter)				1		6	8	NO	no
189	1	1	0.2	0.2	28	c.3259G>A	p.Glu1087Lys	c.5882G>A	p.Gly1961Glu			1			28	28	NO	NO
190	1	2	0.6	1	17	c.1715G>A	p.Arg572Gln	c.2588G>C	p.Gly863Ala	c.2894A>G	p.Asn965Ser	1			14	18	NO	NO
191	1	1	0.6	0.6	11	c.1815G>C	p.Trp605Cys	c.3354C>A	p.His1118Gln	c.2971G>C p.Gly991Arg	c.5077G>A p.Val1693Ile	1			10	12	NO	NO
192	2	2	1.1	1.1	11	c.5603A>T	p.(Asn1868Ile)	c.1538T>A	p.(Val513Asp)	c.5461-10T>C			1		11	11	NO	no
193	3	2	1.8	CF	30	c.634C>T	p.Arg212Cys	c.5882G>A	p.Gly1961Glu			1			6	29	NO	NO
194	1	1	0.8	0.8	24	c.6146delA	p.Lys2049ArgfsTer12	c.5882G>A	p.Gly1961Glu				1		18	26	NO	NO
195	1	1	0.3	0.2	27	c.3113C>T	p.(Ala1038Val)	c.1622T>C	p.(Leu541Pro)	c.5882G>A	p.(Gly1961Glu)	1			27	27	NO	NO
196	3	1	0.6	0.7	10	c.1906C>T	p.Gln636Ter	c.5018+5G>A						1	8	9	NO	NO
197	1	2	0.3	0.3	16	c.3191-1G>T		c.6079C>T	p.(Leu2027Phe)				1		15	16	NO	NO
198	3	2	0.3	CF	18	c.6089G>A	p.(Arg2030Gln)	c.3259G>A	p.(Glu1087Lys			1			18	18	NO	NO
199	2	2	0.9	0.8	13	c.634C>T	p.Arg212Cys	c.5882G>A	p.Gly1961Glu			1			9	14	NO	NO
200	1	1	0.8	0.7	9	c.3449G>A	p.(Cys1150Tyr)	c.1609C>T	p.(Arg537Cys)	c.926C>G	p.(Pro309Arg)	1			7	9	NO	NO
201	1	1	0.6	0.7	11	c.768 G>T	p.(Val256=)	c.286A>G	p.(Asn96Asp)			1			7	11	NO	NO
202	1	1	0.3	0.3	14	c.5882G>A	p.Gly1961Glu	c.2264_2266delTCT	p.Phe755del			1			14	14	NO	NO
203	2	1	1.1	1.1	46	c.2971G>C	p.G991R	c.4538A>G	p.Q1513R			1			NA	48	NO - FU after 9 years	NO
204	1	2	0.2	0.2	56	c.4685T>C	p.Ile1562Thr	c.4685T>C	p.Ile1562Thr			1			54	71	NO - FU after 8 years	NO
205	3	3	1	1	33	c.2587+2T>C	c.2587+2T>C	c.5882G>A	p.Gly1961Glu					1	16	47	NO - FU after 12 years	NO
206	3	2	1	0.9	14	c.4537dup	p.(Gln1513Profs*42)	c.5461-10T>C						1	9	21	NO - FU after 9 years	NO
207	1	2	0	0	57	c.4503G>C	p.Glu1501Asp	c.5715-2A>G	c.6729+21C>T					1	57	72	NO	NO
208	3	3	1.2	1.3	9	IVS6479+1G>A		IVS6479+1G>A						1	7	14	NO	no
209	1	2	0	0	47	c.2588G>C	p.Gly863Ala	c.658C>T	p.Arg220Cys			1			47	48	NO	NO
210	2	2	0	0	65	c.5056G>A	p.Val1686Met	c.3004C>T	p.Arg1002Trp	c.2791G>A	p.Val931Met	1			65	68	NO	NO
211	3	3	1.3	1.5	46	c.2588G>C	p.Gly863Ala	c.5461-10T>C					1		15	52	NO	no
212	2	1	0.8	0.8	7	c.4469G>A	p.Cys1490Tyr	c.6449G>A	p.Cys2150Tyr			1			NA	8	NO	yes
213	1	1	0.7	0.7	9	c.1804C>T	p.Arg602Trp	c.885delC					1		7	7	NO	yes
214	1	1	0.2	0.2	29	c.5196+1G>A		c.5882G>A	p.Gly1961Glu				1		29	31	NO	NO
215	3	3	1	1.3	25	c.1856T>A	p.(Ile619Asn)	c.3210_3211dup	p.(Ser1071Cysfs*14)				1		8	23	NO	NO
216	3	3	1.5	1.5	46	c.5044_5058del15		c.3386G>T	p.Arg1129Leu			1			21	46	NO	NO
217	1	1	0.4	0.4	33	c.53G>A	p.Arg18Gln	c.5882G>A	p.Gly1961Gln			1			8	33	NO	NO
218	1	1	0.5	0.4	42	c.6449G>A	p.Cys2150Tyr	c.5882G>A	p.Gly1961Glu			1			42	42	NO	NO
219	1	1	0.3	1	25	c.5931_5941dup11	p.Phe1982LysfsTer14	c.5882G>A	p.Gly1961Glu				1		22	25	NO	NO
220	1	1	0.2	0.2	41	c.5882G>A	p.Gly1961Glu	c.2297G>A	p.Gly766Asp			1			NA	41	NO	NO
221	1	1	0.3	0.3	36	c.4124C>A	p.Ala1375Glu	c.5882G>A	p.Gly1961Glu			1			34	39	NO	NO
222	2	2	1	1	71	c.4139C>T	p.Pro1380Leu	c.1805G>A	p.Arg602Gln			1			50	73	NO	NO
223	2	2	1	1	32	c.5714+5G>A		c.5461-10T>C						1	9	33	NO	yes
224	3	2	0.9	0.9	9	c.3259G>A	p.Glu1087Lys	c.3259G>A	p.Glu1087Lys			1			7	11	NO - FU after 2 years	NO
225	1	1	0.8	0.8	25	c.3758C>T	p.(Thr1253Met)	c.5882G>A	p.(Gly1961Glu)	c.3364G>A	p.(Glu1122Lys)	1			17	27	NO	NO
226	1	1	1.8	0.4	61	c.4253+43G>A		c.1906C>T	p.Gln636Ter				1		22	61	NO	NO
227	1	1	1.1	1.1	9	c.3210_3211dupGT	p.(Ser1071CysfsTer14)	c.1906C>T	p.(Gln636Ter					1	8	8	NO	NO
228	1	1	0.3	0.4	9	c.5603A>T	p.Asn1868Ile	c.2588G>C	p.Gly863Ala	c.5461-10T>C			1		7	12	NO	NO
229	1	1	0.6	0.3	26	c.1622T>C	p.(Leu541Pro)	c.3113C>T	p.(Ala1038Val	c.5882G>A	p.(Gly1961Glu)	1			18	26	NO	NO
230	2	2	0	0.2	62	c.1906C>T	p.Gln636Ter	c.67-2023T>G						1	47	63	NO	NO
231	1	1	0	0.2	46	c.4254-3T>G		c.5882G>A	p.(Gly1961Glu)				1		43	47	NO	NO
232	1	1	0.6	0.3	27	c.4253+43G>A		c.5603A>T	p.Asn1868Ile	c.3210_3211dup	p.Ser1071Cysfs*14		1		21	27	NO	NO
233	1	1	0.1	0.1	22	c.5603A>T	p.(Asn1868Ile)	c.4469G>A	p.(Cys1490Tyr)	c.6735T>G	p.(Phe2245Leu)	1			20	21	NO	NO
234	3	2	0.6	0.6	10	c.4417C>A	p.(Leu1473Met)	c.5170T>A	p.Trp1724Arg			1			8	10	NO	NO
